# Sequencing the chloroplast genome of a jujube genotype (*Ziziphus jujuba* Mill. 'Fengmiguan') uncovered a 101 bp insertion in the large-single copy region

**DOI:** 10.1080/23802359.2023.2231246

**Published:** 2023-07-06

**Authors:** Shufeng Zhang, Bin Li, Lu Han, Meng Yang, Mengjun Liu

**Affiliations:** a College of Horticulture, Hebei Agricultural University, Baoding, China; b Center of Chinese Jujube, Hebei Agricultural University, Baoding, China

**Keywords:** Chloroplast, Genome sequencing, Phylogeny, Fengmiguan, Jujube, Bioinformatics

## Abstract

*Ziziphus jujuba* Mill., commonly referred to as jujube, is a species of fruiting buckthorn (family Rhamnaceae) that is frequently found across the Shaanxi, Shanxi, and Hebei provinces of China. The ‘Fengmiguan’ variety of jujube, also known as ‘Honey jar,’ is distinguished by its high yield and sugar content, as well as its strong ability to adapt to different environments. In this study, we sequenced and assembled the chloroplast genome (i.e. the plastome) of ‘Fengmiguan’ jujube using a paired-end short-read sequencing technique. The plastome exhibits a quadripartite structure with a total length of 161,818 bp that consists of a large single-copy region (89,427 bp), a small single-copy region (19,361 bp), and two inverted repeats (26,515 bp). The GC content of the plastome is 36.75%. Annotation of the ‘Fengmiguan’ jujube plastome revealed 123 genes, including 79 protein-coding genes, 36 transfer RNA genes, and eight ribosomal RNA genes. Phylogenetic analysis revealed that the ‘Fengmiguan’ variety is closely related to the ‘Bokjo’ variety. Furthermore, we found four variations between these two varieties of jujube, one of which was a 101 bp insertion. Our findings enhance the current understanding of the phylogenetic relationship between different varieties of *Z. jujuba* Mill., which could possibly aid in the improvement of genetic breeding and population selection in jujubes.

## Introduction

Jujube (*Ziziphus jujuba* Mill.) is a species of fruiting buckthorn (family Rhamnaceae) that grows well in cold, temperate, and warm climates, and is one of the most important species of the Rhamnaceae family due to its economic, ecological, and social importance (Liu et al. 2020). Jujube has a long history of cultivation in China and is a popular food product due to its high levels of nutrients, including vitamin C and organic acids (Shan et al. 2019). The ‘Fengmiguan’ variety of jujube, also known as ‘Honey jar,’ is characterized by its small fruit and high sugar content and is thus ideal for fresh consumption or dehydration (Yao and Guldan 2012). In recent years, the chloroplast genomes of several jujube varieties have been sequenced and subjected to phylogenetic analyses (Zhang et al. 2021). However, the chloroplast genome of the ‘Fengmiguan’ variety has not yet been reported; therefore, it is necessary to decode the chloroplast genome of this variety and compare it to that of other jujube varieties or related species. In this study, we sequenced the chloroplast genome of ‘Fengmiguan’ jujube for the first time and determined its phylogenetic position within the *Rhamnaceae* family; this will provide important information about the ‘Fengmiguan’ jujube plastome that will be valuable for genetic breeding and population selection.

## Materials

**Figure 1.**
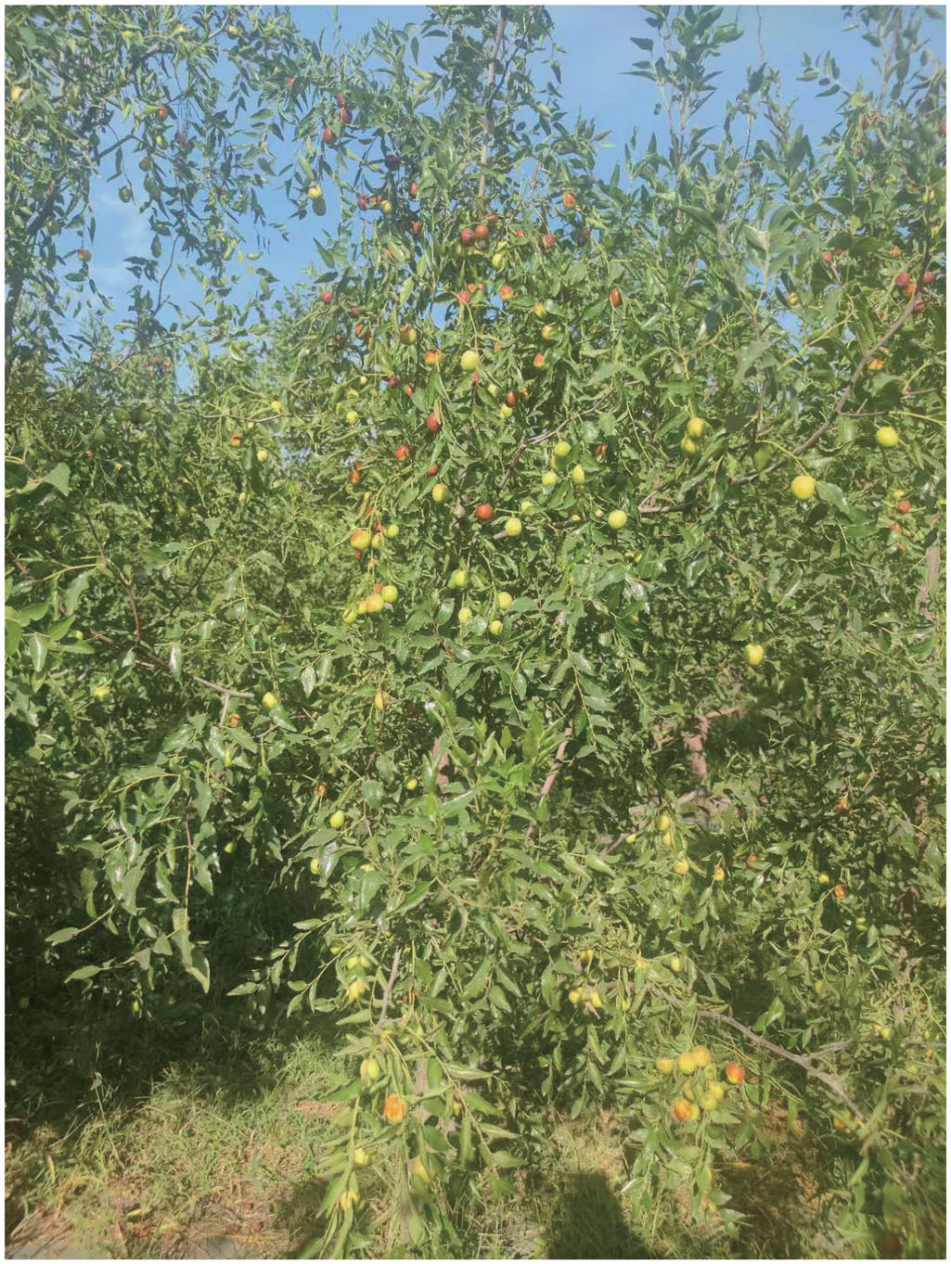
An image of the *Ziziphus jujuba* Mill. ‘Fengmiguan’ variety. This image was taken from the experimental base of Hebei Agriculture University, Baoding, Hebei Province, China.

We collected 30 g of fresh ‘Fengmiguan’ jujube leaves from the experimental base of Hebei Agriculture University, Baoding, Hebei Province, China (115.425°E, 38.815°N, Alt. 79.8 m) (Figure 1). The leaf samples are preserved at the Research Center of Chinese Jujube, Hebei Agriculture University (https://zgzyjzx.hebau.edu.cn/zxgk/zxjj.htm, contact Lu Han, luhan1909@163.com), and DNA samples were stored under the ID number JJB150.

## Methods

### DNA isolation

A modified cetyltrimethylammonium bromide method (Clarke 2009) was used to extract whole genomic DNA from the leaf samples. DNA purity was assessed using a NanoDrop One UV-Vis spectrophotometer (Thermo Fisher Scientific, Waltham, MA), and DNA integrity was verified by agarose gel electrophoresis.

### Library preparation, sequencing, and annotation

The isolated DNA was used for paired-end library construction with an insert size of 200–400 bp, using the official standard protocol, and sequenced on the MGISEQ-2000 platform (BGI, Shenzhen, Guangdong, China). The clean paired-end reads were then assembled using GetOrganelle v1.7.6.1 software (Jin et al. 2020) with the *Z. jujuba* (KU351660) genome serving as a reference. We annotated the resulting chloroplast genome using CPGAVAS2 software (Shi et al. 2019) and manually corrected it using Geneious Prime software (v2021.1.1).

### Phylogenetic analysis

We aligned 31 complete chloroplast sequences using MAFFT (v7.505) (Katoh and Standley 2013) and constructed a maximum-likelihood phylogenetic tree (1000 bootstrap replications; JTT model) using MEGA software (v11.0.13) (Tamura et al. 2007).

### Screening of variant sites

We used msa2vcf software (https://github.com/connor-lab/msa2vcf) to differentiate between single nucleotide polymorphisms and insertion–deletions.

## Results

**Figure 2.**
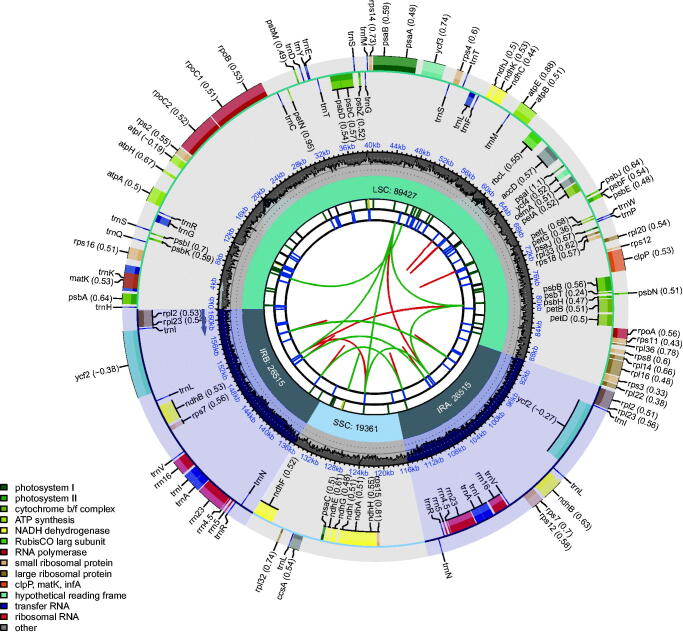
Schematic of the ‘Fengmiguan’ jujube chloroplast genome. The map contains six tracks. From the center outward, the first track shows the dispersed repeats. The dispersed repeats consist of direct (D) and palindromic (P) repeats, connected with red and green arcs. The second track shows the long tandem repeats as short blue bars. The third track shows the short tandem repeats or microsatellite sequences as short bars with different colors. The colors, type of repeat they represent, and the description of the repeat types are as follows: black: c (complex repeat); green: p1 (repeat unit size = 1); yellow: p2 (repeat unit size = 2); purple: p3 (repeat unit size = 3); blue: p4 (repeat unit size = 4); orange: p5 (repeat unit size = 5); red: p6 (repeat unit size = 6). The small single-copy (SSC), inverted repeat (IRa and IRb), and large single-copy (LSC) regions are shown on the fourth track. The GC content along the genome is plotted on the fifth track. Genes are color-coded according to their functional classification. The transcription directions for the inner and outer genes are clockwise and anticlockwise, respectively. The key for the functional classification of the genes is shown in the bottom left corner.

The chloroplast genome of ‘Fengmiguan’ jujube is made up of 161,818 bp and exhibits a quadripartite structure, consisting of a large single-copy (LSC) region (89,427 bp), a small single-copy (SSC) region (19,361 bp), and two inverted repeats (26,515 bp each) (Figure 2). The GC content of the ‘Fengmiguan’ jujube plastome is 36.75% (Figure 2). We detected 123 chloroplast genes, including 79 protein-coding genes, 36 transfer RNA genes, and eight ribosomal RNA genes. Among the annotated genes, only one (*rps12*) exhibited *trans*-splicing, and 10 (*ycf3*, *clpP*, *petB*, *rps16*, *rpoC1*, *ndhB*, *ndhA*, *petD*, *rpl16*, and *rpl2*) exhibited *cis*-splicing.

**Figure 3.**
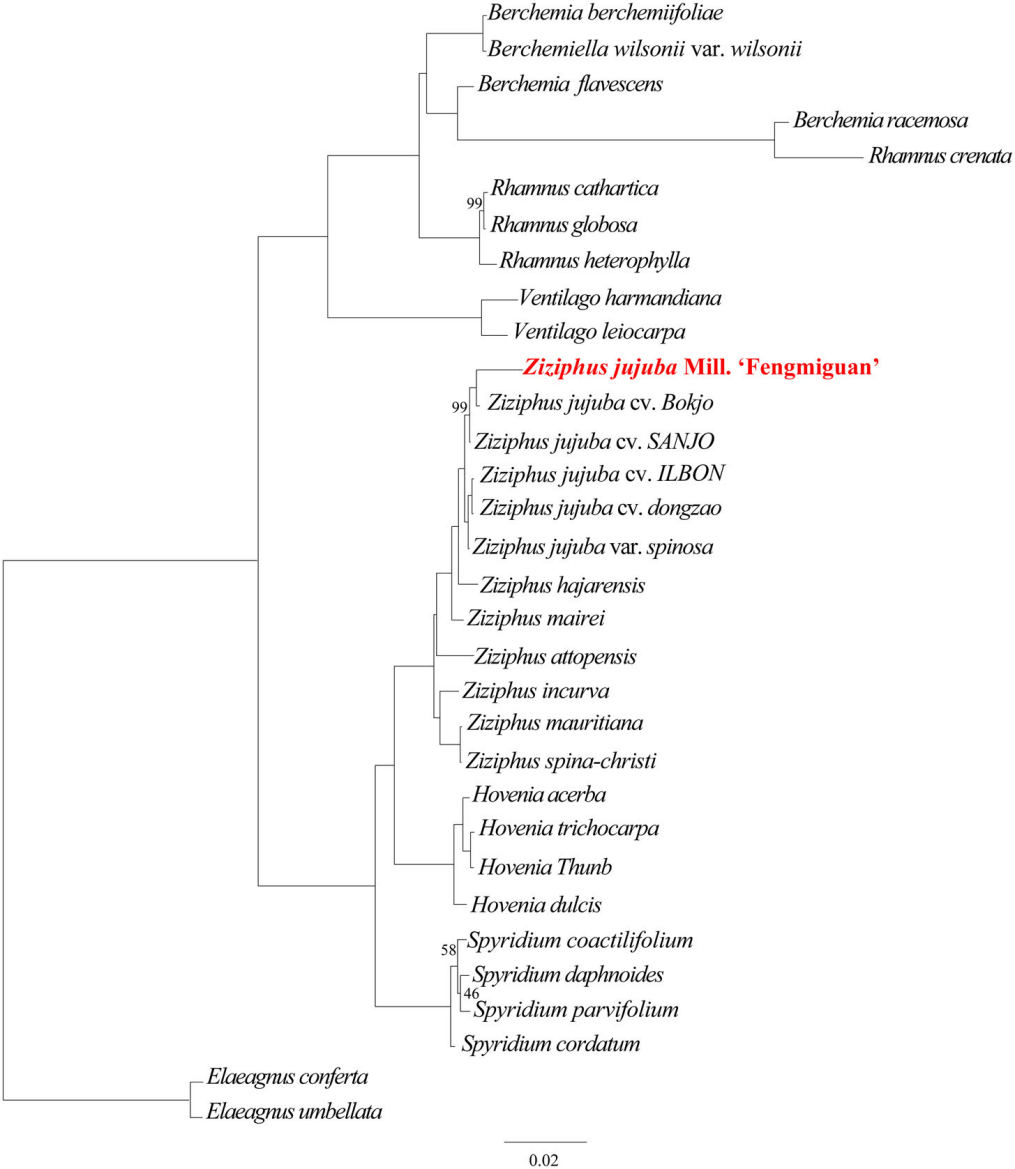
The phylogenetic tree including two outgroups (*Elaeagnus conferta* and *Elaeagnus umbellata*) and one *Berchemiella*, two *Ventilago*, four *Rhamnus*, three *Berchemia*, four *Hovenia*, four *Spyridium*, and 11 *Ziziphus* species. This tree is based on DNA sequences and was constructed using the maximum-likelihood (ML) method. The numbers next to each node indicate the percentage of bootstrap support for 1000 replicates, and all nodes without numbers are 100% supported by the bootstrap sampling test.

To determine the phylogenetic position of ‘Fengmiguan’ jujube within the *Rhamnaceae* family, we performed phylogenetic analysis of the chloroplast genomes from two outgroups (*Elaeagnus conferta* and *Elaeagnus umbellata*) and one *Berchemiella*, two *Ventilago*, four *Rhamnus*, three *Berchemia*, four *Hovenia*, four *Spyridium*, and 11 *Ziziphus* species (Table 1; Figure 3). Phylogenetic analyses revealed that the ‘Fengmiguan’ variety is closely related to the ‘Bokjo’ variety and distantly related to the *Ziziphus mauritiana* and *Ziziphus spina-christi* varieties. Upon comparing chloroplast genomes of the ‘Fengmiguan’ and ‘Bokjo’ genotypes, we discovered four variations within the intergenic region of the LSC region, including two deletions (‘T’ and ‘A’ at positions 34,560 and 54,160, respectively) and two insertions (‘ATTGT’ at position 60,510 and a 101 bp insertion at the 61,067 position). The 101 bp insertion is the primary reason for the difference in length between this ‘Fengmiguan’ genotype and the published ‘Bokjo’ genotype, with lengths of 161,818 bp and 161,714 bp, respectively. Furthermore, we compared the complete chloroplast genome sequences of all five representative jujube genotypes available in the National Center for Biotechnology Information database. Our analysis revealed that this ‘Fengmiguan’ genotype differs from the other jujube chloroplast-available genotypes due to the presence of the 101 bp insertion.

**Table 1.** NCBI accession numbers and citations for the sources of the 31 species used in the construction of the phylogenetic tree.

Species name	Accession number	Reference
*Berchemia berchemiifolia*	MG739656	Cheon et al. (2018)
*Berchemia flavescens*	MK460212.1	Zhu et al. (2019)
*Berchemia racemosa*	ON749761.1	Park and Koo (2023)
*Berchemiella wilsonii* var. *wilsonii*	KY926621.1	Wang et al. (2018)
*Elaeagnus conferta*	MK404307.1	Liu et al. (2019)
*Elaeagnus pungens*	LC522506.1	Lu et al. (2022)
*Hovenia acerba*	MN794429.1	Zhang et al. (2020)
*Hovenia dulcis*	MT916772.1	Liu et al. (2021)
*Hovenia Thunb*	MT225404.1	Li et al. (2020)
*Hovenia trichocarpa*	MN782301.1	–
*Rhamnus globosa*	MT360052.1	Xie et al. (2020)
*Rhamnus crenata*	LC635131.1	Wang and Yang (2021)
*Rhamnus cathartica*	OP374104.1	–
*Rhamnus heterophylla*	MT211599.1	–
*Spyridium parvifolium*	MH234313.1	Clowes et al. (2018)
*Spyridium cordatum*	OK624216.1	–
*Spyridium coactilifolium*	OK624211.1	–
*Spyridium daphnoides*	OK624222.1	–
*Ventilago leiocarpa*	MT974496.1	Lu et al. (2021)
*Ventilago harmandiana*	MZ325585.1	–
*Ziziphus jujuba* var. *spinosa*	MW160433.1	Zhang et al. (2021)
*Ziziphus mairei*	OP480228.1	–
*Ziziphus jujuba* cultivar *SANJO*	MW381776.1	–
*Ziziphus jujuba* cultivar *ILBON*	MW381781.1	–
*Ziziphus jujuba* cv. *dongzao*	MF781071.1	Gao et al. (2017)
*Ziziphus incurva*	MN017132.1	Wang et al. (2019)
*Ziziphus hajarensis*	MZ475300.1	Asaf et al. (2022)
*Ziziphus jujuba* cv. *Bokjo*	MT919946.1	Kim et al. (2022)
*Ziziphus attopensis*	MW201670.1	Li (2021)
*Ziziphus mauritiana*	KY628304.1	Huang et al. (2017)
*Ziziphus spina-christi*	KY628305.1	Huang et al. (2017)

## Discussion and conclusions

In this study, we sequenced, assembled, and annotated the chloroplast genome of one genotype from the *Z. jujuba* Mill. ‘Fengmiguan’ variety. This variety of jujube is famous for its sweet flavor and crisp texture, as well as its abundant fruit production. The chloroplast genome is a suitable model for phylogenetic investigations, due to its specificity (Hu et al. 2022). The comparison of ‘Fengmiguan’ and ‘Bokjo’ plastomes revealed four variations that were present in the ‘Fengmiguan’ plastome, including two deletions and two insertions. These findings contribute to our understanding of the phylogenetic relationship between different *Z. jujuba* Mill. varieties, which will facilitate improved genetic breeding and population selection in jujubes.

## Data Availability

The genome sequence data obtained in this study are openly available in the GenBank of NCBI at https://www.ncbi.nlm.nih.gov/ under the accession number OP537226. The associated BioProject, Bio-Sample, and SRA numbers are PRJNA884017, SAMN31006321, and SRR22243258, respectively.
